# Factors Affecting Referral and Patient Access to Heart Function Clinics in Ontario: A Qualitative Study of Stakeholders

**DOI:** 10.1016/j.cjco.2023.03.002

**Published:** 2023-03-09

**Authors:** Taslima Mamataz, Adeleke Fowokan, Ahmad Mohammad Hajaj, Areeba Asghar, Lusine Abrahamyan, Michael McDonald, Karen Harkness, Sherry L. Grace

**Affiliations:** a Faculty of Health, York University, Toronto, Ontario, Canada; b KITE Research Institute, Toronto Rehabilitation Institute – University Health Network, University of Toronto, Toronto, Ontario, Canada; c BC Centre for Disease Control, Vancouver, British Columbia, Canada; d QU Health, Qatar University, Doha, Qatar; e Institute of Health Policy, Management, and Evaluation, University of Toronto, Toronto, Ontario, Canada; f Toronto General Research Institute, Toronto, Ontario, Canada; g Ted Rogers Centre for Heart Research, Toronto, Ontario, Canada; h Peter Munk Cardiac Centre, University Health Network, University of Toronto, Toronto, Ontario, Canada; i Ontario Health–CorHealth Ontario, North York, Ontario, Canada; j McMaster University, School of Nursing, Hamilton, Ontario, Canada; k Temerty Faculty of Medicine, University of Toronto, Toronto, Ontario, Canada

## Abstract

**Background:**

Though heart failure patients benefit from multidisciplinary care in heart function clinics (HFCs), utilization is suboptimal and inequitable. This study investigated factors influencing referral and patient access to HFCs from multiple stakeholders’ perspectives, namely policy-makers (PM), providers at HFCs and patients.

**Methods:**

In this qualitative study, semi-structured interviews with a purposive sample of Ontario stakeholders were conducted between February-June 2020 and July-December 2022 (paused due to pandemic) via Teams. Interview transcripts were concurrently analyzed using systematic text condensation with Nvivo. Two authors coded individually, with disagreements discussed with senior author.

**Results:**

Interviews with 7 HFCs (6 physicians, 1 nurse), 6 PM and 4 patients were completed before saturation; 5 themes emerged. First, with regard to health system organization, stakeholders reported gaps related to continuity of care, limited capacity and insufficient funding. Second, with regard to referral appropriateness and timeliness, sub-themes related to unclear referral criteria, varying clinic scope, and delays in triage, testing and time-to-visit. The third theme related to clinic characteristics, raised issues of varying clinic services and composition of healthcare professions/expertise. The fourth theme regarding patient factors related to comorbidity/frailty, socioeconomic status, barriers due to location (parking, traffic) and affinity to specific providers. The final theme related to the COVID-19 pandemic concerned increased referral volumes, loss to follow-up care, transition to online delivery modalities and patient refusal of in-person visits. Many facilitators to improve HFC referral and access were raised.

**Conclusions:**

Resources must be provided, and stakeholders brought together to standardize and integrate the HF care continuum.

## Graphical abstract

**Figure:**
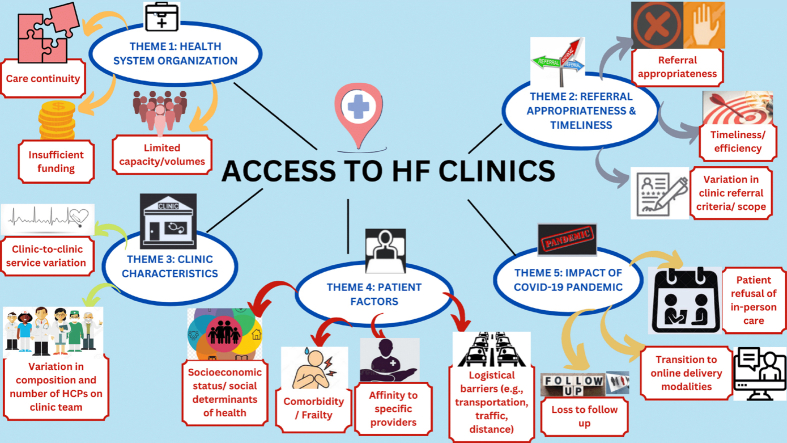

## Résumé

**Contexte:**

Bien que les patients souffrant d’insuffisance cardiaque bénéficient de soins multidisciplinaires dans des cliniques de fonction cardiaque, l’utilisation de ces dernières est sous-optimale et inéquitable. Cette étude visait à examiner les facteurs influençant l’orientation et l’accès des patients aux cliniques de fonction cardiaque du point de vue de plusieurs parties prenantes, à savoir les décideurs politiques, les professionnels de la santé travaillant dans ces cliniques et les patients.

**Méthodologie:**

Dans le cadre de cette étude qualitative, des entretiens semi-structurés ont été menés de février à juin 2020 et de juillet à décembre 2022 (interruption en raison de la pandémie) sur la plateforme Teams avec un échantillon d’intervenants choisis à dessein. Les transcriptions des entretiens ont été analysées simultanément en utilisant la condensation systématique de l’information à l’aide du logiciel Nvivo. Deux auteurs ont effectué individuellement l’encodage, et les divergences ont fait l’objet de discussions avec l’auteur principal.

**Résultats:**

Des entretiens avec des professionnels de sept cliniques de fonction cardiaque (6 médecins, 1 infirmière), six décideurs politiques et quatre patients ont été réalisés avant l’atteinte de la saturation des réponses, ce qui a permis de dégager cinq thèmes. Premièrement, en ce qui concerne l’organisation du système de santé, les intervenants ont signalé des lacunes liées à la continuité des soins, une capacité limitée et à financement insuffisant. Deuxièmement, sur le plan de l’adéquation et de la rapidité de l’orientation, les sous-thèmes étaient liés à des critères d’orientation mal définis, à des champs variables d’application clinique et à des retards dans le triage, les tests et les consultations. Le troisième thème portait sur les caractéristiques des cliniques et les questions relatives à la diversité des services cliniques et à la composition du personnel et des experts en santé. Le quatrième thème avait trait aux patients, notamment leurs troubles concomitants, leur fragilité, leur statut socio-économique, les difficultés d’accès (stationnement, circulation) et l’affinité avec certains professionnels. Le dernier thème découlait de la pandémie de COVID- 19 et concernait l’augmentation du nombre de patients, la perte de contact durant le suivi, la transition vers des modalités de prestation en ligne et le refus des patients de se présenter en personne. La question de la nécessité d’un grand nombre de facilitateurs pour améliorer l’orientation et l’accès aux cliniques de fonction cardiaque a également a été soulevée.

**Conclusions:**

Des ressources supplémentaires sont requises, et les intervenants doivent travailler de concert afin d’assurer un continuum de soins normalisé et intégré pour les patients atteints d’insuffisance cardiaque.

Heart failure (HF) is a chronic, progressive, and complex disease affecting close to 65 million people worldwide.^1^^,^^2^ About 100,000 Canadians are newly diagnosed with HF each year, and 750,000 are currently living with the condition.^3^ As in other high-income countries, despite advancements in pharmacologic and device therapies, the epidemic of HF is expanding alarmingly, with high mortality and readmission rates.^4^ Given that HF has no cure, secondary prevention is the goal; clinical guideline recommendations to reduce disease progression and optimize quality of life are many.^5^^,^^6^ However, achieving optimal medical therapy is challenging for providers for many reasons, including contraindications to therapy, dynamic changes in the clinical status of patients, comorbidities, and inertia.^7^, ^8^, ^9^ Moreover, for patients, achieving optimal self-management is challenging, as it requires sustained health behaviour changes in many arenas (eg, daily medication adherence, weighing, diet, exercise, and symptom monitoring), which must be implemented in the context of psychosocial, cultural, environmental, and economic barriers.

Although their composition and structure varies,^10^ heart function clinics (HFCs) are comprehensive outpatient disease management clinics facilitating rapid care access to prevent acute decompensation, staffed by a multidisciplinary team of subspecialists.^11^^,^^12^ HFCs provide assessment, patient education on self-management skills, medication optimization, and follow-up as needed. Some clinics are more specific to assessment for devices or advanced transplantation candidacy, for example. HFCs have been shown to reduce HF-related mortality by 10%-15%, HF-related hospitalizations by 30%-56% and all-cause readmissions by 15%-25%,^13^^,^^14^ and also to be cost-effective.^15^ Therefore, guidelines from major cardiac societies globally recommend referral to these clinics, although no consensus has been reached on referral criteria regarding which patients would be best served.^16^^,^^17^

Despite the established benefits of HFCs, only approximately 10% of patients receive care from HFCs, and inequities are notable.^18^ For instance, female-identifying and older patients, living in rural areas, and those of lower socioeconomic status receive care less often than others.^16^ Issues related to referral (ie, action required by healthcare providers and clinic staff) and access (ie, action required by patients, such as attending appointments) impede optimal use of these services. These challenges were exacerbated by the COVID-19 pandemic, when access to cardiac care was reduced significantly.^19^, ^20^, ^21^, ^22^, ^23^ A previous review by our group revealed only minimal research investigating why patients are not accessing HFCs.^18^ Moreover, a recent survey of HFCs across Canada recommended the development of explicit patient and risk-based guidance on who should or should not be seen in an HFC (including mode of delivery, which is very germane in the current COVID-19 era).^7^ Therefore, the objectives of this study were as follows: (i) to investigate factors affecting referral and access to HFCs from multiple stakeholders’ perspectives, namely policymakers (PMs), providers in HFCs, and patients with HF; and (ii) to identify facilitators to improving appropriate use.

## Methods

### Design

This qualitative study was informed by an 8-member expert panel comprised of a patient organization, an HF administrator, HF physician subspecialists, an HFC provider, members of leading HF committees in the country, a scientist with content expertise, and a methodologist. The study was approved by the institutional review boards of University Health Network (CAPCR ID#19-6171) and York University, Toronto. All participants provided written informed consent. Interviews were conducted in February–June 2020, and were then halted due to the COVID-19 pandemic. Interviews resumed in July 2022, and continued through December. The study was reported in accordance with the Consolidated Criteria for Reporting Qualitative Research (COREQ) guidelines,^24^ and best practices to ensure the rigor of the qualitative methods were followed.^25^

### Setting and participants

The study was conducted in Ontario, Canada, where healthcare delivery is under provincial jurisdiction. Ontario has an estimated 36 HFCs.^22^ Each clinic serves a median of 200 patients per year, with an estimated 2000 annual patient visits. Overall, 157 HF physicians and 60 nurse-practitioners are providing care in these clinics. However, clinic services vary, with less than half offering implantable defibrillator or cardiac resynchronization therapy expertise, and only one-tenth have expertise in heart transplant or mechanical circulatory support. In addition, although most clinics optimize guideline-directed medical therapy along with medication and dietary consultation, remote monitoring and community partnerships for home visits are still very limited. Nevertheless, advanced care directives and end-of-life planning discussions are offered in most of these clinics.^10^^,^^26^^,^^27^

Three stakeholder groups were included and purposively sampled, namely the following: Ontario PMs and administrators (eg, Ministry of Health, Health Quality Ontario, CorHealth Ontario [now Ontario Health]; heads of major divisions of cardiology); healthcare providers currently working in HFCs (eg, physicians, nurses); and patients with HF (including both those who did and who did not access clinics). Participants were interviewed until theme saturation was achieved.

PMs are those who plan, organise, direct, and coordinate health services. For recruitment of Ontario PMs, CorHealth Ontario’s Cardiac Hospital Administration Committee members were contacted. HFCs were identified through a previous environmental scan^26^ and were contacted through our expert panel members.

HF patient participants were reached through our patient partner organization—the HeartLife Foundation (https://heartlife.ca/)—social media, and the Ted Rogers Centre for Heart Research’s Heart Hub. Patient inclusion criteria included living with HF in Ontario and having English-language proficiency. Those with significant cognitive impairment, or a lack of willingness to have the interview recorded, were excluded from this study.

As interviews proceeded, expert panel members were asked to identify potential interviewees with characteristics that differed from those of participants (eg, different types of institutions, professions, sex). For HF patients, attempts were made to recruit both males and females, and to have representation of patients living within and outside of urban areas.

### Procedure

Semistructured interviews were conducted through the Teams platform (Microsoft, Redmond, WA); face-to-face interviews were avoided, in consideration of COVID-19. Potential interviewees were e-mailed an invitation to participate; nonresponders were contacted again 2 weeks later. A reminder e-mail was sent to the interviewees a few days before the interview, including the interview questions.

The interview questions were shared on screen throughout the interview, and all parties had their cameras turned on during the process. Interviews were audio-recorded, and video-recorded so that nonverbal communication could be considered in analyses. Facial expressions, hand gestures, tone of voice, and pauses were noted in each interview. Interviews were led by a senior member of the team (S.G. or L.A.), and a trainee observed the interview and took notes (A.F. or T.M.). Interviews were approximately 45 minutes in length.

### Materials

To capture the diverse array of perspectives by stakeholder type, a separate semistructured interview guide was designed for each type. The interview guides were developed based on our reviews of the literature^16^^,^^18^ and information on HFC care in the province.^10^^,^^27^ Input from the expert advisory panel was solicited and incorporated into the final version of the interview guides (see Supplemental Appendix S1). When data collection resumed in 2022, questions about the impact of the pandemic on HFC access were added to the interview guides.

### Analyses

First, each interview transcript was cleaned, to ensure accuracy and anonymity. Then, data coding was performed individually by 2 authors (T.M., A.H., A.F., or A.A.) concurrently with data collection. The data were analyzed using NVivo version 12 (QSR International, Burlington, MA). Given that 3 different stakeholder groups were involved, transcripts were analyzed using systematic text condensation.^28^ Each transcript was read thoroughly, to obtain a general impression. Then, the meaning-bearing units that described the same central meaning were identified by reviewing each transcription systematically. Next, a codebook was developed based on extracted meaning units, with constant comparison applied to identify, expand, and merge themes across the stakeholder groups.^28^ All codes were subsequently read through and analyzed for similarities and differences across participants and stakeholder groups. This process was followed by a reconciliation meeting held to review and come to agreement on the coding for each transcript, as well as the text condensation. Any disagreements were reconciled through discussion with the senior author (S.G.). The identified themes and subthemes—illustrated by quotes from the interviewees—were then reviewed by the expert panel for confirmation, which adds credibility to the findings.

## Results

**Figure 1:**
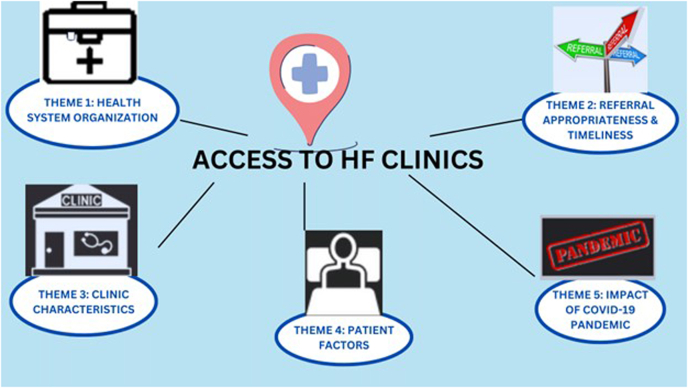
Five major themes on access to heart function clinics. HF, heart failure.

A total of 17 interviews were conducted before saturation was achieved; 5 were conducted post-pandemic. Characteristics of the 7 HFC providers, the 6 PMs, and the 4 patient respondents are shown in Table 1. Analysis revealed 5 themes, with associated subthemes, as illustrated in Figure 1. With the addition of the COVID-19 question, an additional theme was identified; not many other responses differed before vs after the pandemic. Exemplary quotes are shown in Supplemental Table S1. Facilitators to address identified challenges are shown in Table 2.

**Table 1:** Characteristics of interview participants, by stakeholder group

Characteristics	n (%) or median (range)
*Patients*	4 (23.52)
Sex, female	2 (50.00)
Age, y	34 (20–65)
Geography/residence	
Urban	3 (75.00)
Other	1 (25.00)
Duration living with HF, y	5 (2–11)
Recent visit to emergency department for HF, yes	4 (100.00)
*Heart function clinics*	7 (41.17)
Profession	
Physician	6 (85.71)
Nurse	1 (14.28)
Years worked at clinic	16 (2–30)
Institutional type	
Tertiary centre	5 (71.42)
Other	2 (28.57)
Number staff working	9 (7–30)
Annual patient volume / caseload per clinic	800 (400–2200)
*Policymakers*	6 (35.29)
Jurisdiction	
Provincial	1 (16.66)
Regional or other	5 (83.33)
Education	
Master’s	5 (83.33)
Doctorate	1 (16.66)
Years working in cardiac care policy	11 (3–32)

HF, heart failure; y, years.

**Table 2:** Facilitators to referral and access to HF clinics as perceived by stakeholder groups

Theme	Facilitator
Health system organization	Department/committee meetings across continuum of care, to track patient flow and timeliness/wait for 1st HF clinic visit
	Triage tool for the emergency department
	Facilitating relationship between tertiary and community HF clinics, to extend capability of community clinics. Examples: • Spoke–hub–node CorHealth Ontario policy∗ • Augmenting capacity of Family/Ontario Health Teams • Leverage cardiac rehabilitation programs in communities without HF clinics
	Electronic referral, leveraging electronic medical records
	Training/education of emergency physicians regarding clinic availability and services
	Linkage with nursing homes
Referral appropriateness and timeliness	A one-stop online resource for referring physicians to characterize HF severity and act accordingly. Examples: • COACH trial^35^ • CorHealth Ontario’s HF Care Support tool†
	Clear referral criteria (eg, the I-NEED-HELP‡ acronym from the American College of Cardiology Expert Consensus Decision Pathway for Optimization of HF Treatment^36^)
	Marketing, communicating referral criteria
	Rapid-access clinics^35^
	Foster relationship with diagnostics departments
	Intra-clinic triage
Clinic factors	Clinic relationship with emergency and inpatient departments at hospital, so can support shorter length of stay, which is hospital goal for reimbursement reasons
	Linkage with referring providers and other providers in circle of care (eg, give them information they need to manage patient [while ensuring good communication about medication changes], etc.)
	Linking with other HF patient care resources (eg, palliative, community / home care [weighs, blood pressure assessment])
	Protocol for nonphysician/nurse to handle patient symptoms and medication changes by phone, so clinic has capacity for new patients
	Electronic dashboard tracking referrals, current patient load, and discharge eligibility
Patient factors	Protocol for nonphysician/nurse to handle patient symptoms and medication changes by phone so patients do not need to travel in to clinic
	Offering virtual appointments
	Patient-oriented discharge summary
	Appointment reminders (interactive voice response)
	Engaging family supports, informal caregivers

COACH, **C**omparison of **O**utcomes and **A**ccess to **C**are for **H**eart **F**ailure; HF, heart failure.

∗ https://www.corhealthontario.ca/resources-for-healthcare-planners-&-providers/integrating-heart-failure-care/The-Spoke-Hub-Node-Model-of-Heart-Failure-Care.

† https://www.corhealthontario.ca/resources-for-healthcare-planners-&-providers/integrating-heart-failure-care/Overview.

‡ I-NEED-HELP is an acronym for **I** = **I**ntravenous inotropes; **N** = **N**YHA class IIIB/IV or persistently elevated natriuretic peptides; **E** = **E**nd-organ dysfunction; **E** = **E**F ≤ 35%; **D** = **D**efibrillator shocks; **H** = **H**ospitalizations > 1; **E** = **E**dema despite escalating diuretics; **L** = **L**ow systolic BP ≤ 90, high heart rate; **P** = **P**rognostic medication; progressive intolerance or down-titration of GDMT.

First, with regard to health system organization, subthemes of gaps in continuity of care, limited capacity, and insufficient funding were raised. With regard to the former, respondents spoke to the importance of timely identification and diagnosis of HF patients by a primary care provider, who then would refer the patient to an internist or cardiologist. The specialist then would refer the patient to a HFC, as appropriate. Emergency and primary care physicians must be aware of HFC availability as well, to support appropriate and equitable referral of patients.^29^ With regard to capacity/volumes, clinics should be located based on the regional incidence of HF, with capacity and expertise to match the needs of the population. Although location is becoming less important with the increased availability of virtual care, some visits should be conducted in-person. Moreover, a wide variation was present in terms of capacity of each HFC, and in their approach to managing referrals when they were at capacity. Variation in clinic approach to discharging patients was also found; some clinics kept patients through to end-of-life, whereas others kept them for 1 year or until they were stable or fully titrated on medical therapy, to create capacity for new patients. Finally, with regard to funding, the main issue was the lack of a way to bill directly for clinic services other than physician time. Thus, the funding and resources (eg, overhead, administrative support, nurses) to run the clinic were coming from the global hospital budget, which often was insufficient and perceived as undependable.

Second, with regard to referral appropriateness and timeliness, subthemes were related to unclear referral criteria, and varying clinic scope, as well as delays in triage, testing, and time-to-visit. Referral criteria/scope varied widely by clinic, and often were dependent upon the expertise of the physicians. As outlined earlier, many required a patient to be seen first by a cardiologist; other clinics based acceptance on number of hospital visits, medication factors (including adherence), or need for advanced therapies, for example. In many cases, these criteria were not explicitly stated—either at the clinic, or with the referring clinicians; this lack of explicit criteria often resulted in receipt of “inappropriate” referrals, whom many clinics then spent time redirecting elsewhere. In addition, there was lack of consensus on what the clinic referral criteria should be, with some clinics tightening or changing criteria over time to reduce unmanageable referral volumes, again often without targeted communication to the referral base. With regard to appropriateness, many clinics had the perception that they are not receiving referrals for those patients who are most in need, but they have not had the ability to test this view directly. The timeliness/efficiency of referral processes were also perceived as deficient. Clinics were aiming to reduce re-admissions, particularly with the government focus on reducing the 30-day rates, thus patients need to be seen well before 30 days from hospital discharge. However, clinics often receive incomplete referrals, creating delays in assessing appropriateness for HFC services as well as determining priority.

The third theme related to clinic characteristics, raised issues of how variation in clinic services and composition of healthcare professions/expertise impacted patient referral. As outlined earlier, clinics varied in the information they required to consider and accept patients. They also had different modes of accepting referrals, with the availability of more options being preferred. Services also varied from clinic to clinic; for example, some clinics focused on candidacy for devices or advanced therapies, whereas others focused on more general aspects of HF care, such as medication titration. With regard to the latter, the nature and number of the healthcare providers also varied. Some clinics had 1 physician, whereas others had many, who were specialists or subspecialists. Others had a mix of nurses and specialist nurse-practitioners. The nature of the allied healthcare complement and administrative staff varied as well, all impacting the number and type of patients who could be seen, and when. Finally, the clinics also varied in how they covered physician cancellations, again with an impact on access to care.

The fourth theme regarding patient and social factors, identified subthemes were comorbidity/frailty, socioeconomic status, barriers due to location, and affinity to specific providers. For instance, some patients had physical limitations, necessitating accompaniment by a caregiver. Clinic location had implications for proximity to home/travel time and conditions, parking cost and availability, as well as traffic density. Patients preferred providers who had shorter wait times—both for an appointment date, and on the day of the appointment (ie, they wanted the provider to be on time). Some patients lacked primary care providers or preferred the subspecialty care at the HF clinic to that of their cardiologists and/or referring physicians, so they were pursuing care based on preference for a provider/bedside manner, rather than appropriateness.

The final theme related to the COVID-19 pandemic with subthemes concerned were, increased referral volumes, transition to online delivery modalities, and loss to follow-up care, exacerbated by patient refusal of in-person visits. Some patients were trying to avoid the healthcare system for fear of contracting the virus, or could not get an appointment to see primary care providers. A related issue is that clinics reported receiving more referrals, many of which were not appropriate. Clinics reported that patients or caregivers refused necessary in-person visits on some occasions; these included situations in which informal caregivers were not vaccinated, or did not want to mask, and hence were not allowed into a clinic with infection control policies requiring these. In cases in which virtual appointments were appropriate, the capacity to treat these patients hinged on the technological capability of not only the clinic, but also the patient, in terms of available hardware and devices, software, technical support, and verbal communication skills. Many older patients joined the virtual appointments with support of their adult children.

## Discussion

This study was the first to investigate multilevel factors in referral and access to HFCs in a public health system, including during the COVID-19 pandemic.^30^ This study was conducted in Ontario, Canada, where it is urgently needed, as indicated by a recent report from the auditor general. The report highlights a lack of full implementation of the recommended HFC community model across the province, despite its demonstrated benefit in several regions.^31^ The major themes, which coalesced across the multiple stakeholders interviewed, were health system organization-related challenges, referral appropriateness and timeliness, variation in clinic characteristics, patient-related factors, and the pandemic (Fig. 1).

Consistent with quantitative surveys of HFCs and reviews in the country,^10^^,^^16^ the major challenges to an optimal continuum of care for HF patients appear to be the following, a lack of regional coordination of care at the government level; the limited number of clinics and capacity of existing clinics; lack of organization, standardization, and clarity regarding the purpose and specialization of clinics given the existing variation^10^; the lack of formal communication channels across the continuum and circle of care; and lack of guidance on who should or should not be referred to HFCs. Consistent with the literature on access to other outpatient chronic disease care,^32^^,^^33^ as well as prospective Canadian studies^34^ and reviews on HF clinic access specifically,^18^ the patient-related barriers identified were related to social determinants of health, their health status, transportation (parking cost, traffic, distance), time, and technology.

Some conflicting viewpoints and needs were expressed by stakeholders. For example, patients sometimes refused in-person visits for fear of contracting COVID-19, or were unable to enter a site because the informal carers accompanying them were not in compliance with COVID-related policies. However, based on types of diagnostic tests, length of time since seeing a patient, or level of risk, providers often need to have in-person rather than virtual visits. Tension was also present relating to balancing the need to reduce variation in HFC capacity and approach while also matching these to the needs of the population, particularly given the diversity in Ontario.

Many facilitators for improving HF care in the community and reducing the need for acute care were identified (Table 2). CorHealth—which promotes the “spoke-hub-node” model—is an important mechanism to support coordination across the continuum of care and among HFCs, to facilitate better care coordination, standardization, efficiency, and patient-centredness, even in the COVID-19 context. This model suggests that level of care and setting should be based on patient risk and complexity, from “spokes”—for stable, low-risk patients to receive care in the community—to tertiary “nodes” where high-risk patients with complex needs receive care in an advanced cardiac hospital. In an HFC network in the province, the “node” level of the recommended “spoke-hub-node” model was in place, but it ceased, due to insufficient support. The system needs connection to primary care “spokes” and “hubs” in a fully regional model, and that connection is explored in some of our forthcoming work. Again, given the population and geographic diversity in the province, the standardized model should be resourced and implemented based on regional needs.

Standardized, evidence-based recommendations regarding who should be referred to HFCs are also needed. The I-NEED-HELP acronym (see Table 2) from the American College of Cardiology Expert Consensus Decision Pathway for Optimization of HF Treatment is an example of a such a recommendation for advanced patients. Canadian guidelines provide some direction as well (see Table 2 of^16^). Moreover, the **C**omparison of **O**utcomes and **A**ccess to **C**are for Heart Failure (COACH) trial undertaken in the province is a promising model for the care continuum.^35^ The intervention comprised a point-of-care algorithm that stratified HF patients based on risk of death, to support hospital discharge decisions, but an important aspect was that this was coupled with rapid follow-up in HFCs for those discharged. Some interviewees were part of the trial, and they greatly advocated for the model (Table 2), which would also improve referral appropriateness and timeliness. With results demonstrating significant 30-day reductions in mortality and morbidity with the rapid-access care, implementation should be pursued. Finally, clinic staffing and funding policy should be re-visited, so they can be resourced to provide a full cadre of needed care in a patient-centred manner.

The results of this study have implications not only for policy, but also for future research. While many HFC access facilitators were identified, the expert panel perceives it would be premature to develop guidance until the viewpoint of those who refer patients to HFCs, namely primary and acute HF care providers, are also sought, and until evidence regarding appropriate but also feasible HFC inclusion and exclusion criteria is undertaken (eg, test the I-NEED-HELP acronym from the American College of Cardiology Expert Consensus Decision Pathway for Optimization of HF Treatment^36^). With this information, an expert panel could be convened to undertake a formal, evidence-based process to develop recommendations on improving the HFC system.

Caution is necessary when interpreting the results. First, representative generalizability is not established through qualitative research, so although purposive sampling was used and saturation was achieved, the applicability of the results to other provinces or healthcare systems cannot be known. Another point to note is that the patient population did not have representation from rural areas, and included only a few health care professionals (HCPs) working at nontertiary centres. Second, although face-to-face interviews are ideal, interviews were performed via videoconference, given the COVID-19 pandemic. However, one member of the research team notated nonverbal communication during each interview. Finally, the nature of the study design precludes causal conclusions.

In conclusion, this qualitative study gleaned the perspectives of PMs, HFCs, and patients regarding gaps in referral and access to HFCs—gaps that impede optimal care quality and hence quantity of patient life—in a public healthcare system. The main themes identified were related to health system organization, referral appropriateness and timeliness, clinic-related factors, patient-related factors, as well as the COVID-19 pandemic. It is hoped that these findings, congruent with quantitative and other local evidence, as well as the recent Auditor General’s report, will spur consideration of care alignment with CorHealth Ontario’s regional model of integrated care and the recent COACH trial findings. Resources must be provided, and stakeholders brought together, to standardize and integrate the HF care continuum, so that patients who need HFC care most will access and benefit from such care.
