# Supplementary material for: Factors Affecting Referral and Patient Access to Heart Function Clinics in Ontario: A Qualitative Study of Stakeholders
Source: CJC Open. 2023 Mar 9;5(6):421–8. doi: 10.1016/j.cjco.2023.03.002 (PMC10314100; doi:10.1016/j.cjco.2023.03.002)
Supplement: Supplementary Data [file mmc1.pdf]

Supplemental Appendix S1.

Interview questions (Policy-makers/Administrators)

1. Let's just start with a few questions about your role if you are willing to provide that for context.
    a. What is your jurisdiction in terms of cardiac care (institution(s), province…)
    b. Tell me about your position and what you do in relation to HF clinics
    c. What is your education / training background?
    d. How long have you worked in heart policy /practice? (years)
2. What policies has your organization created or are planning to create regarding HF clinics and outpatient HF care?
    a. Does your organization work from association or other organization's policies on HF clinics? If yes, which ones?
3. What is the current status of HF clinics in your jurisdiction from a system perspective?
    a. Number, who should access …
4. When thinking about HF care, what role does your organization envision for HF clinics?
    a. Is this different than current reality?
5. In your jurisdiction, do you think the appropriate patients access HF clinics?
    a. Why or why not?
        i. Who should access them less vs more?
6. What kinds of policies / tools do you think are needed to optimize use of HF clinics in your jurisdiction?
    a. e.g., toolkits/protocols/checklists aimed at standardizing the referral process for all clinics?
    b. policies aimed at improving referral rates to heart failure clinics?
    c. Tracking and feedback to EDs?
7. Do you have any departments/teams or committees/ working groups focused on HF clinics in your organization?
    a. Are there regular opportunities to discuss HF clinic organization and performance in your jurisdiction?
        i. What are they? Who sits at these tables? What is generally discussed?
            1. Is there enough time and energy do you think to consider the organization of HF clinics and their role in the outpatient care journey?
8. COVID-19 has greatly impacted acute and chronic care. In what ways do you perceive it has hindered and / or facilitated HF clinic utilization?
9. Do you have any final suggestions on how access and referral to HF clinics could be optimized that we haven't covered?

Interview questions (Heart failure clinics)

1. Let’s just start with a few questions about your role if you are willing to provide that for context.
    - What is your profession?
    - How long have you worked at the HF clinic?
    - Where is your clinic located? (ie.., tertiary care ctr, community)
    - How many staff work at the clinic?
    - How many pts do you estimate you treat / year?
2. What do you think are the mechanisms or processes by which patients are referred to your clinic? Please describe.
3. In your opinion, do you think the referrers do a good job at referring patients to your clinic?
    - Appropriate patients (e.g., acuity), timeliness
    - Are there patients you think you should be seeing but do not get referred for instance?
4. What do you do with referrals that are inappropriate / do not meet your referral or inclusion criteria?
    - Probe: communication with referring doc, other docs in circle of care, patients
5. COVID-19 has greatly impacted acute and chronic care. In what ways do you perceive it has hindered and / or facilitated HF clinic access for patients?
    - Probe: faster implementation of remote care technologies to support patient care in the community
6. For patients who do not show at their initial or miss appointments, what are some common reason why they are unable to attend?
7. What policies or organizational-related factors negatively affect patient referrals and access to your clinic? Please describe
8. What barriers have you encountered in receiving patient referrals?
    - Any facilitators?
9. Do you have any final suggestions for improving the referral system or access for appropriate patients that we haven’t discussed?

Interview Questions (Patients)

1. Let's just start with a few questions about you if you are willing to provide that for context.
    a. Sociodemographic
        i. Age, sex, size of area lived in [urban, suburban, rural]?
    b. Clinical:
        i. Do you know what type of HF you have, or approximately what is your ejection fraction?
            1. e.g., HFpEF or rEF
        ii. How long have you had it?
        iii. Have you gone to the emergency department for your HF?
2. How would you describe a heart function clinic?
    a. PROBE
3. Could you describe your experience with being referred to a heart function clinic?
    a. What did you remember about it?
    b. Where were you? Were loved ones present?
    c. Who told you about the referral?
        i. Doctor, nurse…
    d. Did they tell you why you were being referred?
        i. If yes, what did they tell you?
    e. Were you provided any pamphlets to take home about the clinic or referral?
    f. Did they tell you the next steps like whether you should call the clinic and the number or when to expect a call from the clinic?
    g. What do you think worked well? What didn't work well?
4. Do you think there is anything your healthcare providers could have done to make it easier to access the HF clinic?
    a. Please describe.
5. Do you think your doctor provided you with enough information to know what you were being referred for and why?
    a. If not, what else did you want to know?
6. Did you actually go to the HF clinic?

If yes:

- Did you face any barriers going to the heart function clinic? If yes, could you please describe?
- Are there any factors or actions that were taken by the doctor that helped facilitate the transitioning to the specialist clinic?
- Were you provided with adequate information before your appointment to prepare you for your visit at the HF clinic?
- Did the clinic provide you with a time and date that was convenient for you?
- Did you go more than once?

    a. Did ever miss an appointment at the HF clinic? What was the reason?

If no:

- Are there any personal factors (e.g. financial, family, health (comorbidities) etc.) that made it difficult for you to attend the specialist clinic?
    a. Parking costs, transportation, distance

7. Please share any final comments related to your overall experience with the referral system that we may have missed.

Supplemental Table S1: Exemplar Quotes by Sub-Theme

**Theme** (Sub-theme)	Quote (participant type, number)
**Health System Organization**	
Care continuity	In short, the system in the province is poorly structured to manage HF patients. …It was usually like eight weeks or 12 weeks or something like that, for the family doctors to follow-up… and the worst-case scenario was … there was no follow-up. (PM4)<br>Yeah, I do feel that, because these are the kind of, these are the same patients that keep coming into our Emerg. because they're not being properly managed, or they don't know where to go and so they don't have anywhere else to go [such as a clinic]. They come back to Emerg. (PM5)<br>We have an established HF pathway that looks towards ensuring that patients have follow-up in our HF clinic within one to two weeks post-discharge…. Currently, we only see about half of the patients that we discharge with a diagnosis of HF. And we know that if we're not seeing the majority of them, the risk for them to be readmitted to the hospital due to a HF complication is much higher. (PM6)<br>My cardiologist decided because, after almost nine years I'm stable, XX is much closer for us to go to. And I also see, my electrophysio cardiologist at XX and I also go to XX for my pacemaker defibrillator. So, I already go to XX for two different cardiology issues. Yeah, so she felt that I might as well go there for the HF clinic. But I haven't been accepted there yet, so I don't know. So, I was referred in October to XX regional health center and I didn't hear anything. (PT1)<br>So, … on discharge summaries for example, there's a note that says 'refer to HF clinic'. And nobody's picked up the referral. (HFC1)<br>We have no streamlined way of working through, um, orders from a telephone perspective. So, I know a lot of other hospitals will work on titration of medications like Lasix and potassium and sometimes beta-blockers, depending on what patients are showing clinically on the outside. And a lot of that is just a call and they have to maintain parameters within a certain range for blood work, et cetera. And we don't really have a standardized way of doing that. And it's very physician-oriented… it puts a bit of a hitch in the plans of smooth transitions to the community, and they end up coming back fairly frequently for clinic visits that might have been avoided otherwise, and our population's not very mobile. (PM1).<br>The plan is basically for faster inpatient discharge and stronger community supports for patients with HF that are recognized as having HF and meeting certain eligibility criteria, um, to follow-up with that. …It seems to be very physician dependent; Some patients we are seeing early enough that we can start them on medication, that we see an improvement in their EF, follow-up [echocardiograms], et cetera. And then the other patients we see, it's too many hands in the pot trying to change things; those people

	need to be seen more regularly by at least one clinic that can look after everything. (PM1)<br><br>So, we are looking for policies, like how do we enable and utilize Family Health Teams and how do we engage primary care and general practitioners so that they know enough, that they're knowledgeable enough to support their patient with HF so that they're not solely relying on a clinic. What's our role as a regional hospital to support the smaller hospitals in maximizing their efforts in supporting health for the patients? (PM2)<br>We identified that the pathway for an HF patient wasn't clear, especially in the acute care setting and where they go. And what we identified is that even in the city of [xx] we had 16 different discharge destinations that a patient with HF could utilize post-discharge from an acute care hospital. (PM2)<br>The government is focused on the 30-day readmission rate. If you think about it, if a patient's being discharged from hospital, but no one sees the patient, or many patients aren't seen within 30 days. And I often say if you don't see that patient until the 29th day post-discharge, you're not going to reduce 30-day readmission. So, there are huge challenges, uh, in the system as well. (PM4)<br>In hospital at the time of discharge, they'll just put a discharge order for follow-up and HF clinic, and then that comes electronically to us. And then the other route is just like paper-based, like fax referrals. (HFC7)
Limited capacity/ volumes	HF clinics have capacity issues, you know, broadly…We're working through the hub-and-spoke model to be able to increase the capacity of some of the smaller hospitals … and we're doing that through virtual clinics and through virtual visits with patients and families. (PM3)<br><br>One of the issues is capacity… I think we probably could be better at discharging patients from our HF clinic. If we go back to what I was talking about earlier about, you know, the goal to prevent readmissions, we're not able to see all patients after HF hospitalizations. And part of that is because often patients get stuck in their HF clinic. (HFC7)<br>The big one is lack of capacity in the community, which is big…..There's not enough capacity to manage HF in the community and we can't do it all…These are people living in the community and if their HF could be managed -- especially when they're more stable --more often in the community to prevent exacerbations, then obviously we'd be able to see more patients coming in on the other end. (HFC3)<br>Yeah, I think there's some, there's some areas, there's certainly some regions that don't have access to any kind of clinic. They're being seen as part of a primary care office, um, practice. And when they run in to trouble, they'll refer them to other centers. But there's some areas that don't have a HF clinic in their community at all. (PM3)<br>But I think part of the challenge in terms of referrals is also discharging from the program. And that gets back to the, um, capacity of the system to

	look after the less complex, more stable HF patients. There's lack of comfort in doing that. And it's sort of a bottleneck really. (HFC3)<br>So, there's always this, um, tension between who is eligible for a HF clinic and who isn't. And it depends on volumes. (PM4)
Insufficient funding	There's no funding for a HF clinic right now. …. If a hospital sets up a HF clinic, they're doing it and pulling it out of their global budget or they've done some other gymnastics to take out some money. (PM4)<br>You know, there are silos in funding in the system, and the patient suffers. Often the people who administer such programs see the world from their biased siloed perspectives, right? And so, um, the hospital has a budget and a lot of income from hospitals comes from surgeries and MRIs and procedures. HF is not a money-maker for anyone… So, to me, the way the system is funded does not reflect the needs of people with chronic disease. (HFC3)<br>So, a patient comes into hospital, they might get admitted, and then they get discharged. They have no follow-up. They get put on some low dose of medications and you know, maybe we'll see them [in clinic] in six months, 'cause we get paid better for that. So, it all has to do with billing. (HFC4)<br>For funding from an administrative perspective, a HF patient lives in the community. They generally have multiple other problems, and the way that funding is allocated on a regional basis is, is disproportionately hospital based. And so, from the funding perspective, if you want to increase capacity in the community, you know, an engineer or a business person will say, well, you shift money from acute care to the community-- which you can't do because they're funded through separate envelopes. (HFC3)<br>So as you are aware, the government doesn't pay for clinics. It's paid for by the hospitals, and the region…So this clinic here is run by the hospital. So there are very strict criteria because they're looking for a very specific goal. The goal was to decrease hospital readmissions... we're not seeing people who are otherwise the walking well, we're seeing the sick.. Yeah, that was the original reason. Otherwise, the hospital, was very interested, but they had no money. (HFC4)<br>But I think, the advantage of the hospital is that you know, there's a nurse and you know all those things that are kind of paid for by the hospital. So if we are to set up an HF clinic at our private clinic, where do we get the funding to support that? (HFC7)<br>If I put my hat on and said, okay, I'm going to sit as an administrator for the government. I could say, well, I don't know that I'm getting value for my money. Like you tell me, describe HF clinic. What's the structure of a HF clinic? And I would say, no one knows… We need to say that this is a HF clinic, so if you have X, Y, and Z and you have the population base, yes, then we will fund you to run a HF clinic. And I'm not talking about paying doctors, I'm talking about the infrastructure, which would include the administrative support, the nursing support. (PM4)

	Because the patient population is large, and I think that's the key issue. So, you define what is a heart failure clinic, what are the components of a heart failure clinic and what are the goals of the heart failure clinic and then you fund it appropriately…It doesn't have to be in the university center, it could be anywhere, but you need to meet certain criteria and they would fund that. The Ministry is not interested in anything that's open-ended; They want very clear, defined things. (HFC4)
**Referral Appropriateness and Timeliness**	
Variation in clinic referral criteria / scope	And then I go over each of the referrals with their supporting data to say whether or not they should be seen by us. A lot of times I feel that and say they should at least be seen by a cardiologist first before seeing us. So, we are a second step sort of clinic… Obviously, if they're very sick, we'll see them without having a cardiologist at first. But we prefer them seeing a cardiologist; Have at least follow-up and tried to do some initial HF therapies. (HFC5)<br>Well, there's not enough capacity to manage HF in the community and we can't do it all. And so, you know, that's partly why the [referral] criteria are a bit stricter. (HFC3)<br>I think the idea of let's open up the doors, everybody, it's very ideal but you won't be able to cope with them and you won't be able to discern between who's sick and not. (HFC4)<br>But we, as far as acuity goes in area and all of that, we don't put a limit on where we're receiving patients from… And we've had patients that have come from other clinics that no longer want to be followed there and would rather come to us. So it's sort of built around preference in that perspective (PM1)<br>Well, I think because we're the largest tertiary clinic in the [XX], well, we are the only tertiary center, it's part of our mandate to be accessible. (PM2)<br>So if you don't treat people then it won't be very long before they meet the criteria, right? A lot of patients, we do a very good job with them because a lot of patients were under-treated, and you know, there's exceptions to the rule. (HFC4)<br>And we see patients primarily on just one day a week, which is a little bit of a limitation. And we don't really turn away anybody, although the patients primarily come from inpatient… But we would never turn any family doctor, any referral from anywhere away. (PM1)<br>I mean, uh, we don't [have referral criteria]. As I said, we don't really use checklists or anything... I mean, I think it's just sort of like our gut feeling that this patient could use some more support [such as] what we can offer in the clinic. (HFC7)<br>If you say, well, I'll take them in after one hospitalization. In a year, you're getting less sick individuals, the volume goes up. But, again, if you say, okay, we will take people into the HF clinic who have had two hospitalizations within the last year, um, you realize that you're getting sicker patients, but you're ignoring all the less-sick patients. So, there's

	always this trade-off. I think the HF clinics are overwhelmed right now. So, to say we're all going to open up the tap and pour more patients into them. All we're going to do is end up generating longer wait lists. In fact in [xx] we've re-evaluated our inclusion criteria and tightened it up. (PM4)<br>And because these are high-intensity HF patients, so we have a very strict criteria for coming to our clinic. (HFC5)<br>So each clinic is probably a bit different or, you know, more lenient vs more restrictive based on their capacity or things like that. But I don't think that we're too strict in terms of, you know, who gets to get into the clinic. (HFC7)<br>We're missing a good chunk of the population by not seeing the cardiac-oncology population; we have to refer those patients elsewhere… The ones that have had HF just cause they're on chemo. I think a lot of the, the issue is the role of the clinic isn't understood across the board by all hospitalists, and about who should access right away. (PM1)
Referral appropriateness	Unfortunately, education is one of them, like these inappropriate referrals, it's because the referring physician is not educated exactly on what heart failure is. (HFC5)<br>So in terms of meeting criteria, they generally do sort of. I guess the question is, are all patients who should be referred, referred? And I don't think so. Just so you can avoid the re-admissions that obviously plague HF patients. Mainly a lot of frail, older people tend to be not seen as worthy. (HFC3)<br>I think if somebody's being referred and the cardiologist feels that that they're not needed in the clinic, they would probably have a conversation with the referring physician. Sometimes they'll just say they want a plan for medication up-titration, so they can ask for some very specific things. But we would always be back in touch with the referring physician if we thought for some reason that, that the patient was inappropriate. (PM3)<br>Um, because there are patients who are low-risk and there are patients who are high-risk. And the question was, can we improve the triaging of those patients? (HFC2)<br>We can speculate, if you look at the literature, that often a general practitioner does not actually recognize patients who actually have HF and would benefit from sub-specialty HF care, whether that be in the community or tertiary. (HFC2)<br>As far as the family doctor referral process, I think it's just, if they recognize it and they see it, they'll refer. There's nothing standardized….Um, but there's no standardization of where patients are sent. (PM1)<br>So we do get referrals that are not 'classic' HF. So I'm not sure if you would call them inappropriate referrals, but we do see patients that are sort of outside the realm of general HF…. I think all clinics feel that there's general HF that is not being referred. So, a lot of us do see that. But I'm not really quite sure how to, uh, substantiate that. (HFC2)

	Well, all referrals are screened as they come in. And then they are prioritized based on the referring clinicians' indication. We can redistribute, so if a patient is referred that does not appear to be a HF patient, we may redistribute that, so that we don't use the HF clinic resources, but they're still seen. (HFC2)<br><br>We've got a lot of patients that end up there that have exacerbated COPD but they haven't had a BNP, and they get referred to us and it's not an appropriate referral. (PM1)<br><br>Well, the Nurse-Practitioner will help …redirecting those patients to the appropriate environment. (PM6)<br><br>So depending on what the patient presentation is, I either redirect the referral to our general cardiology clinic that's outside of the hospital or if they've been seen by a cardiologist, which is sometimes the case, I'd redirect back to the cardiologists and say you can follow-up on this concern. Or if it's completely inappropriate, we just sent back to the GP or whoever it was that referred, and say 'no', that this is inappropriate; I would say less than 10% we just send back without some sort of action. We actually do the work to re-refer them back to other clinics. (HFC5)
Timeliness / efficiency	I think a lot of the issue is the role of the clinic isn't understood across the board by all hospitalists, and about who should access [one] right away. So, we have some physicians that are great at referring from the inpatient side and others where we see patients repeatedly get admitted before it's finally made. So, it's really variable because I think a lot of it is very physician dependent, unfortunately. (PM1)<br>With the ones that are referred, are they referred on time? Like is it at the right time? Sometimes, no. (HFC3)<br>Yeah, it seems to be the limitation is in terms of how soon we can see them. Um, just because we're built around just the two physicians that have limited time in clinic. So that's the limitation. (PM1)<br>Sure. I mean these clinics are very busy for sure. And um, you know, we do watch, I mean you have to watch how long the gap in time is between patient visits and make sure it's timely. (PM3)<br>A lot of it is just the support that the HF clinic can provide for the inpatient end. And about how fast they can safely discharge and how fast we can triage patients, 'cause there's always a shortage of spots. (PM1)<br>In terms of records, for a lot of these referrals we have to dig through multiple different systems, as I don't think they use [the same electronic health system]. So if we need notes, we have to figure out who the clinician is and ask their office directly. So it's a lot of time to process these referrals, because you wanna know all the information before you spend the time to sit down and talk with them in-person and whether or not it's appropriate. So, our nurses have to dig a lot because yeah, because the clerical staff might not know whether the patient is relevant or not. So, then it takes the nurses to go hunt and look. And so more time and energy. (HFC5)

	When I talk to physicians about why they choose to send to us, a lot of it is wait time; So, if they see shorter wait times elsewhere, they'll send patients elsewhere. (PM1)<br>We can accept every patient, but the waitlist is long. I would say we have a fairly good compliment right now of general cardiologists. It's just because of the demand, the wait period is as long. (PM2)<br>I feel like the funding and the wait time for HF clinic might need to improve, like, uh, I didn't wait quite long to go into clinic. I feel like either solving that problem with an intermediate solution or having quicker access to clinic is really important….. And you know that physical touch point is super, super important… Um, but without the occasional touch points, it felt very long. … I got pretty lost most of the time. (PT2)<br>It can extend out as far as a month, sometimes up to six weeks if it's really bad. (PM1)<br>So for example, if we found like the readmissions were up in the hospital part, we might take a look at the clinic and see whether or not people are getting timely access to clinic resources, et cetera. (PM3)<br>It's really just, uh, trying to see people. And we really do try to see people no longer than one month waiting time. And, you know, we originally started at two weeks, which became a little bit impossible. But we're pretty well on to one month, and that's again because we're very strict with the criteria and stick to those criteria. (HFC4)<br>It's variable depending on what the on-call schedule is for those those physicians. So for instance, March is a really bad month for them. That might look more like a three to four-week interval, but we have other periods of time where they can be seen within seven to 14 days. So, I would say if you want to put an average on it, anywhere between two to three weeks, but it can extend out as far as a month, sometimes up to six weeks if it's really bad. (PM1)<br>…but when it comes from the community though, they'll ask 'what's your wait time for seeing this patient next'? And if we're shorter than the next place they were going to refer to, then they'll refer to us, and if we're too long then they'll refer them downtown. (PM1)<br>Well there's a big fear of the wait time in terms of seeing patients. (PM1)
**Clinic Characteristics**	
Clinic-to-clinic service variation	I think that it's very heterogeneous. One of our priorities is to be accessible. So it does mean that we have a bit of a reputation for also seeing challenging or unusual or strange cases as well. (HFC2)<br>So this HF clinic at [XX] was designed to be a discharge HF clinic, meaning it's specifically for patients who have just been discharged from hospital or have gone to the emergency room because of what was felt to be HF as the primary diagnosis. So that's the intended population. We see these patients for three to six months, during which time our goal is to optimize their HF, medical therapy, sort out the etiology of their HF. The intention of this clinic was specifically to see patients who have just been

	discharged from hospital. That's its mandate. (HFC6)We are not sophisticated like Toronto, but we have different sorts of clinics….. So some of those patients may go to the pre-transplant clinic. (HFC4)<br>And there seems to be a lot of variability in terms of the palliative component. We right now link with [palliative clinic]. I know the central LHIN has got a strong link with their clinical nurse consultants for their hospice and palliative care. They seem to be bigger centers. (PM1)<br>As i said, we all have specific expertise. So we have expertise in hemodynamic assessment and cardiac biopsy and autonomic physiology. So those referrals are sometimes from out-of-province as a result. (HFC2)<br>They have like a dedicated slot where they see OTN patients through basically webcam interviews that are linked with nursing homes, and they'll have this beautiful setup where it's the nurse-practitioner in clinic seeing the patient through webcam; a dedicated time every week. (PM1)
Variation in composition and number of HCPs on clinic team	A lot of concerns because we ended up where our clinic is staffed with two nurses right now. We were staffed with three, and two physicians. (PM1)<br>The current model is it's one afternoon a week on Mondays and it's 10 patients in that clinic on average; three to four new and six follow-ups. That's kind of the balance… it's too much, to be honest. HF patients are very heavy. No, not many people I know do a whole 8:00 AM to 4:00 PM clinic of HF. At least not in academic centers. Nobody would. It's too much. Most clinics are either half a day in the morning or half a day in the afternoon. (HFC6)<br>The second group would be complex patients, with other medical problems that might contribute to their HF, for example, renal failure. As I said, we do have a nephrologist and it's, uh, a patient on dialysis, uh, some of the cancers. So, we work with the lymphoma group, multiple myeloma group. So those sorts of things versus the very specific other illnesses, which HF is a part of. (HFC4)
**Patient Factors**	
Comorbidity/frailty	Some of these elderly patients with mobility issues who are, you know, mostly, you know, home-bound. It's just difficult for them to come to appointments. (HFC7)<br>We had a patient who was quite elderly, over 90, who was not mobile…unable to come. (HFC6)<br>The common reasons they're unable to attend, usually the commonest reason, is that they are too sick to attend. Virtually all of the patients we saw were high-risk. And they were high-risk because of age and frailty… So, we do seem to actually embrace the frail patients. (HFC2)
Socioeconomic status / social determinants of health	Yeah, parking costs are definitely kind of painful… it must be pretty difficult for other people financially to even make these trips to the hospital. (PT2)<br>But when I have to go down to the hospital, I don't have a car, so I have to Uber. So it's expensive and no one's reimbursing me for that. And I'm not working right now. So it's kind of a pain. (PT3)

	And you know, there's algorithms for prescribing. So why can't we have an algorithm for seeing a patient? But the thing is patients are all different, and that gets back to their comorbidities, socioeconomic considerations as well as their disease. (HFC3)
Logistical barriers (e.g., transportation, traffic, distance)	Like I can wake up, my parents have to wake up pretty early and we have to take the day off or something. And then, you know, me being a whole day out of it for the appointment downtown. (PT2)<br>The fact that they need to, you know, get someone to transport them over. So that definitely makes them miss appointments a lot of times. Getting the message to them… sometimes the phones aren't working. We're kinda a little archaic that we still sometimes mail out our appointment times. But again it you know they don't get the mail or they don't check it so they don't show up the last month. (HFC5)<br>We're notorious; our cardiologists take a while to see patients. Um, so sometimes between seeing the nursing staff and having their full assessment and the cardiologists, they are waiting for like an hour or two hours. A lot of the patients that really hate the wait time just refuse to come back because of that. So it's not to do with clinical status at all. It's just the wait time. It doesn't work for them. (PM1)<br>We have just in the last year have seen an explosion in our referrals and in our heart function census. So we, we have up in over 600 rostered patients in the clinic now. We would like to have more of a regional program where we have satellite clinics within the area because our LHIN is quite large. We have quite a long distance in between our sites. And so it makes it difficult for patients to access care. So we are trying to figure out ways to be able to reach patients in further distances. (PM5)<br>..was how could we utilize technology, remote monitoring software so that patients that are living in [XXX] for example, don't have to travel into [YYY]…. From what we're hearing, the patients would rather be at home. (PM2)<br>So many patients don't want to come to a downtown clinic. Just the issues that come along with being at a downtown clinic such as parking, and just access. So really location would be the major issue that I think would reduce not only referral, but that patients may actually request referral elsewhere closer to home. (HFC2)<br>It was just the distance to [xx] and the traffic. Sometimes if there was some issue on the 401 highway or whatever, we would be late for an appointment. So we would leave quite early in the morning. If we had a nine o'clock appointment, we would leave just after five in the morning. (PT1)
Affinity to specific providers	But a lot of people do wanna be followed by the same people who took care of them right after their hospitalization. So that's where I foresee a bottleneck… not every patient wants, you know, it's very hard to discharge patients. And part of that is because, in my experience, during the pandemic, many of them want to hold on to specialist care. (HFC6)

	And a lot of them will make specific requests to be seen solely by us. (PM1)<br><br>Um, our hospitalist almost always works with the family doctors in the community. A lot of our patients prefer to see her over there. She's like hyper-involved with patient care and they really love that. Um, so a lot of times they'll see her instead of the family doctor routinely. (PM1)<br><br>And part of it I think is that they just sort of like the attention and care that they get. I mean, they have a nurses cell phone number that they can call 24/7, essentially. And so some patients, you know, do become quite dependent on the service that they get and their HF clinic and when we try to discharge them to our general cardiology clinic they resist that. (HFC7)<br>We've had patients that have come from other clinics that no longer want to be followed there and would rather come to us. So, it's sort of built around preference in that perspective. (PM1)
**Impact of COVID-19 Pandemic**	
Transition to online delivery modalities	I'm hopeful that we're going to see more. It is a little more challenging with our HF population as they are a much older demographic. However, quite often those patients, their children are involved in their visits, and we see that as helping to facilitate that move towards virtual clinic visits. And actually we were getting sort of a trial by fire right now with, with the whole COVID situation. You know, today's a perfect example. We're seeing a few urgent patients today in our clinic this morning, but the cardiologist is conducting the rest of his visits over the phone. (PM6)<br><br>Now we did move a lot more to telephone or divert some of the follow-ups. (HFC5)<br><br>We kind of functioned pretty much throughout all of COVID with very minimal virtual visits because of the type of patients we take care of; because I can't talk to somebody on the phone until I really understand what their volume status is. (HFC6)<br><br>The thing that I know has come up recently is physician compensation through the use of, um, technology or telemedicine. There's some restrictions that Ontario is starting to put on through OHIP for the way the physicians get funded (PM2)<br>We currently run a virtual clinic, um, for about, I'd say 20% of our existing patient population. So, if they have a clinic appointment that won't involve any diagnostic imaging or lab tests, they have their appointments set-up over OTN as long as they are able to carry that out. (PM6)<br>I think we got a lot more referrals where they should have been seen by someone else first… The family physician never even assessed this patient in-person, and then was just sent to us. (HFC5)
Patient refusal of in-person care	I think from a practical level, it did get a bit difficult in terms patients refusing to come in because they were worried about COVID, unfortunately then end up in the hospital… They refused to come see us, we couldn't do anything… It was harder because we couldn't communicate with the care partner because the care partner refused vaccination. (HFC5)

	I think like COVID has, I mean particularly you know providing HF-based care in hospital-based clinics, I think COVID definitely threw a wrench in that… a lot of the patients were, like, terrified to come into hospital. (HFC7)<br>With COVID, like a lot of reluctance from the patient's standpoint and some of that has lingered as well… Yes, they were afraid of catching COVID. (HFC7)
Loss to follow-up	But because of COVID, I at the moment don't have a cardiologist. I've been discharged from one hospital and the other clinic is not taking anybody… I called the HF clinic, and I asked and they said don't expect to hear from the HF clinic for months because of COVID. So I don't even know if I've been accepted… I feel like I'm in limbo at the moment. It's very, it's stressful in that sense. (PT1)<br>We were looking into the fact that we miss about 50% of our patients, but at the same token as well, um, the question is, okay, if we saw all of a sudden had all of those 50% coming in for appointments, how would we manage that because it would quickly become unmanageable. So part of the issue with that is our workflows are slowed down due to COVID. (PM6)

BNP, Brain Natriuretic Peptide; COPD, Chronic Obstructive Pulmonary Diseases; COVID, Coronavirus Disease; EF, Ejection Fraction; Emerg., Emergency department; PT, patient; PM, policy-maker; HF, Heart Failure; HFC, Heart Function Clinic; HCP, Health Care Professional; LHIN; Local Health Integration Network (i.e., regional health authority); MRI, Magnetic Resonance Imaging; MD, Medical Doctor; NP, Nurse-practitioner; OHIP, Ontario Health Insurance Planning; OTN, Ontario Tele-health Network;
